# The Virtual Neurologic Exam: Instructional Videos and Guidance for the COVID-19 Era

**DOI:** 10.1017/cjn.2020.96

**Published:** 2020-05-21

**Authors:** Mariam Al Hussona, Monica Maher, David Chan, Jonathan A. Micieli, Jennifer D. Jain, Houman Khosravani, Aaron Izenberg, Charles D. Kassardjian, Sara B. Mitchell

**Affiliations:** Division of Neurology, Department of Medicine, Postgraduate Medical Education, University of Toronto, Toronto, Ontario, Canada; Division of Neurology, Department of Medicine, St. Michael’s Hospital, University of Toronto, Toronto, Ontario, Canada; Department of Ophthalmology and Vision Sciences, and Division of Neurology, Department of Medicine, University of Toronto, Toronto, Ontario, Canada; Florida Medical Clinic, Wesley Chapel, Florida, USA; Division of Neurology, Department of Medicine, Sunnybrook Health Sciences Centre, University of Toronto, Toronto, Ontario, Canada; Neurology Quality and Innovation Lab (NQIL), University of Toronto, Toronto, Ontario, Canada; Department of Psychiatry, Sunnybrook Health Sciences Centre, University of Toronto, Toronto, Ontario, Canada

**Keywords:** Neurological examination, Virtual care, Telemedicine, COVID-19, Physical examination

## Abstract

**Objective:**

To outline features of the neurologic examination that can be performed virtually through telemedicine platforms (the virtual neurological examination [VNE]), and provide guidance for rapidly pivoting in-person clinical assessments to virtual visits during the COVID-19 pandemic and beyond.

**Methods:**

The full neurologic examination is described with attention to components that can be performed virtually.

**Results:**

A screening VNE is outlined that can be performed on a wide variety of patients, along with detailed descriptions of virtual examination maneuvers for specific scenarios (cognitive testing, neuromuscular and movement disorder examinations).

**Conclusions:**

During the COVID-19 pandemic, rapid adoption of virtual medicine will be critical to provide ongoing and timely neurological care. Familiarity and mastery of a VNE will be critical for neurologists, and this article outlines a practical approach to implementation.

## RÉSUMÉ

**Effectuer un examen neurologique à distance : instructions vidéo et orientations à l’ère de la pandémie de coronavirus (COVID-19)**.

**Objectif:**

Mettre en évidence les caractéristiques d’un examen neurologique pouvant être effectué à distance au moyen de plates-formes de télémédecine; fournir des orientations pour passer rapidement d’évaluations cliniques en personne à des « visites virtuelles » en ces temps de pandémie de coronavirus (COVID-19), voire à plus long terme.

**Méthodes:**

Seront décrits en détail les aspects d’un examen neurologique complet qu’on peut aborder à distance.

**Résultats:**

La présentation d’un examen neurologique de dépistage mené à distance sur une grande variété de patients de même que des descriptions détaillées d’actes nécessaires selon des scénarios spécifiques (tests cognitifs, examens de troubles neuromusculaires et de troubles du mouvement).

**Conclusions:**

En ces temps de pandémie de coronavirus (COVID-19), l’adoption rapide de la médecine à distance est essentielle si l’on veut offrir des soins neurologiques en temps opportun et de façon continue. Le fait d’être familier avec les examens neurologiques à distance et de les maîtriser deviendra donc crucial pour les neurologues. Voilà pourquoi la présente étude entend mettre en relief une approche pratique permettant la mise sur pied de tels examens.

## Introduction

The COVID-19 pandemic has necessitated rapid and immediate changes to the way physicians deliver care. Nonurgent, face-to-face visits have all but stopped, and yet we must endeavor to provide high-quality and timely patient care.^1,2^ A home video visit (HVV) is one technology allowing physicians to perform new or follow-up assessments through telemedicine, incorporating video and audio interactions.

The urgency to pivot to virtual care during the pandemic poses many challenges, one of which is adapting the neurological examination to a virtual interface.^3,4^ The examination is an essential component of the neurological consultation, but few, if any, neurologists are trained to perform a virtual neurologic examination (VNE). The discomfort toward the VNE may be a barrier to general and subspecialty neurologists adapting HVV into practice, in turn influencing patient access to neurologists during the COVID-19 pandemic and beyond.

The purpose of this document and accompanying videos (see Supplementary Material) is to describe a VNE that can be performed on most, if not all, patients (Table 1), along with further details and maneuvers targeting specific clinical presentations (Tables 2 and 3). These recommendations are critical during this period of rapid HVV implementation, but remain useful beyond the ongoing pandemic, as the VNE can be used to assess patients who live at a distance or in underserved communities.

**Table 1:** Suggested screening VNE

Mental status	Orientation: State date. Language and recent memory: Ask a question about recent events (e.g., pandemic). Assess fluency of speech and any obvious receptive or expressive aphasia or confusion. Attention: Count backward from 100 by 7’s.
Cranial nerves	Pupils: Observe for symmetry then reaction to light by having the patient cover and uncover each eye independently. Eye movements: Look in the nine cardinal positions of gaze with brief pause at each position. Saccades: Alternate gaze between upper right and left corner of screen, and then just above and below the screen. Facial strength: Lift eyebrows, squeeze eyes shut, show teeth, and purse their lips, observing for any asymmetry. Speech: Comment on dysarthria or dysphonia. Neck flexion: Turn head right and left and then shrug shoulders. Tongue: Observe the tongue at rest for bulk and fasciculations, then stick out tongue and move side to side.
Motor exam	Assess muscle bulk in upper and lower limbs Observe for abnormal movements in the limbs Assess for pronator drift and forearm rolling As a basic assessment of symmetric antigravity power, have the patient move through a full range of motion in both upper and lower limbs Perform 10 body-weight squats and unilateral heel raises (can be performed with gait assessment)
Sensory	Specific regions to test depend on reason for referral and sensory complaints (e.g., assessing a specific peripheral nerve distribution). As a general screen: Ask the patient to compare light touch (or cold using ice) on the index fingers of both hands and the top of the big toes.
Coordination	Rapid-alternating, finger-to-nose (or finger-to-object), and heel-to-shin movements Bradykinesia testing with finger tapping and opening/closing fist
Gait	Observe stance and ability to stand with feet together Observe gait, and ability to walk in tandem

**Table 2:** Expanded mental status exam

MoCA by telephone	The blind version of the MoCA can be downloaded from MoCA website (https://www.mocatest.org) with visual elements removed, scored out of 22 (normal cutoff ≥19).^8^
MoCA with audiovisual Conferencing	Full explanation at https://www.mocatest.org. In brief, patient requires paper and pencil to complete visuospatial/executive/ language component. Can be facilitated by showing patient the prompts via camera or screen-sharing options.
Executive and visuospatial function	Clock drawing task:^9^ Draw a clock, put all the numbers in, and set the hands at “ten past eleven.” Note clock contour, number positioning, hand placement, and any visuospatial neglect. Phonemic and semantic fluency: Name “F” words in 1 min. Compare with semantic/category fluency (name animals in one minute). Luria test: Patient to place their hand on a flat surface and then alternate movements between fist-palm-side.
Memory	Repeat and remember three words, and then test recall after a 5-min delay. Use category or multiple-choice cueing if necessary.
Language	Naming: low- and high-frequency objects such as thumb and knuckles. Repetition and comprehension: Single-word repetition followed by a longer phrase such as “the lion was eaten by the tiger” and then ask “which animal is still alive?” to determine grammatical comprehension. Reading and writing: Reads a sentence provided and then writes a sentence spontaneously.
Praxis	Pantomime certain actions (e.g., brushing teeth) for ideomotor apraxia, and check for orobuccal involvement by having them pretend to blow out a candle. Note body part as tool, temporal or spatial errors.

**Table 3:** Specific scenarios

Myasthenia gravis	Sustained (1–2 min) upgaze test for fatigable ptosis or extremes of gaze for diplopia. Curtain sign: Ask patient to lift more ptotic eyelid and look for the less ptotic lid to become more ptotic. Fatigable limb weakness: Hold arms in the air for 120–240 s, and while supine holds each leg up for 120 s at a time, looking for fatigability.
Movement disorders	Look for restricted vertical gaze, hypometric saccades, hypomimia, and hypophonia. Observe at rest for any involuntary movements or abnormal postures. Tremor: Assess at rest, with hands outstretched (postural), and with finger-to-nose testing (kinetic). Archimedes spiral can be attempted, or the patient can be asked to pour water from one cup into another. Rest tremor can be brought out with distraction (e.g., saying the months of the year backward). Give a writing sample to assess for tremor or micrographia. Bradykinesia: Open and close fist fully and repetitively, one hand at a time; tap the finger and thumb repetitively at large amplitude; supinate and pronate forearm repetitively; tap each foot and heel on the floor repetitively; reduced arm swing and stride length on gait testing. The Unified Parkinson’s Disease Rating Scale (UPDRS) has been validated using a modified remote version, without rigidity and retropulsion testing.^10^
Stroke	The National Institutes of Health Stroke Scale (NIHSS) is a focused exam, well suited to virtual care in the context of telestroke systems, and can be reliably performed with smart devices during virtual visits.^11^ During the COVID era, those performing a virtual stroke assessment should use appropriate personal protective equipment, and during a hyperacute stroke presentation, a protected code stroke framework should be utilized.^12^

Not all parts of the neurologic examination can be performed virtually, and the VNE cannot entirely replace an in-person examination. In situations where the clinician is concerned about a serious or potentially harmful neurologic condition, alternative means must be sought to ensure that the patient is assessed urgently in-person. Other limitations of the VNE include the inability to perform a detailed power examination (such as for a neuromuscular consultation), fundoscopy (such as for a headache consultation), and neuro-otology maneuvers (for a vertigo consultation), among others.

## A Word about the Neurologic History

It is often taught that the history is the most important element in a neurological assessment and one should have a good sense of the diagnosis (or a short list of differential diagnoses) after obtaining the history. We therefore stress the importance of a detailed and thorough neurologic history, and its weight further increases during a HVV. Details on the neurologic history are beyond the scope of this publication.

## The VNE

### General Comments

The VNE is best performed in a standardized fashion, with the patient seated in a chair in a well-lit room, without visual obstructions between the patient and camera. An open room is ideal, so that the patient can move closer or farther from the camera during the examination, with space to observe gait. Although privacy is an important consideration during an HVV, having a family member or other third party present can aid with certain aspects of the examination including holding the camera. It is important that the assessment be performed through a secure online platform that is compliant with local privacy acts. The patient may be asked to verify their identity by providing their address or date of birth or showing photographic identification. Consent to perform the exam virtually should be obtained and documented. A translator should be present (either a family member or formal third-party translation services) if required.

Later, we provide a detailed outline of the full neurological examination, with commentary on maneuvers that can be performed virtually. Table 1 outlines a streamlined screening VNE that can be used as a framework for most patients. See the accompanying videos for demonstration of these techniques (Supplementary Material).

### Mental Status Examination and Cognitive Testing

Please refer to Supplementary Video 1, for demonstration of a screening virtual mental status examination.

Begin by noting the patient’s level of alertness. Note any bradyphrenia, expressive or receptive aphasia, confusion, or inappropriate affect.

In most cases, a detailed cognitive examination is not required, but as a screen we suggest the following. Orientation can be examined by assessing place (city) and time (day, date, month, and year). The patient should be asked to look straight ahead at the camera while answering orientation questions so as to avoid the use of environmental cues (phone, calendars, and so on). To test attention, instruct the patient to state the months of the year backward or subtract backward from 100 by 7’s.

To screen fluency and recent episodic memory together, ask a question about current events (e.g., pandemic) and then note appropriateness of the answer along with the fluency of speech, the presence of phonemic or semantic paraphasic errors, or the presence of apraxia of speech. Next have the patient name one high- and one low-frequency word (such as knuckles and thumb, respectively). Test single-word repetition by having the patient repeat a word such as “mama,” and then test sentence repetition by having the patient repeat a sentence such as “no ifs ands or buts.” Next have the patient follow simple commands to test comprehension, such as “close your eyes” and then more complex tasks such as “point to the surface that you walk on.” Table 2 outlines an expanded cognitive assessment that can be used if clinically warranted.

### Cranial Nerve Examination

Please refer to Supplementary Video 2 for demonstration of a screening virtual cranial nerve examination.

#### Cranial Nerve 1

This nerve is seldom examined, and abnormalities of smell, such as anosmia, can be screened from the history.

#### Cranial Nerve 2

Formal visual acuity (VA) assessment cannot be reliably performed through video, but problems with VA can be screened from the history. In addition, a simple screen for symmetry of VA between the eyes could be attempted as follows. Ask the patient to locate a book or newspaper and read the same word using each eye separately while it is held at the same distance from their face. Note that this does not provide a formal acuity value. If assessment of vision is critical, the patient can be referred to a recognized website that provides printable Snellen charts, or a Snellen chart can be mailed to the patient.^5^ If there is asymmetry between eyes, the patient can be asked to produce a small hole in a piece of paper to see if vision improves. Color vision can be assessed for red desaturation in each eye using any red object (e.g., pen), or using Ishihara color plates (either held up to the camera or through a smartphone application, although standardized electronic versions are lacking).^6^

A reliable assessment of visual fields is not possible through video, although computer-based or tablet-based fields are available and have shown good correlation with Humphrey visual fields.^7^ The examiner may be able to screen for a gross field cut (such as a marked hemianopia) by asking the patient to position their face about 2 feet away from the monitor while looking at an object held by the examiner (e.g., pen) and then ask the patient if they can see all four corners of the monitor. This technique is only appropriate with a computer monitor or tablet (not a smartphone), and will still not adequately assess for deficits in the extremes of the peripheral fields. Pupillary constriction can be assessed by having the patient cover one eye and then uncover, followed by the other eye and watching for the appropriate response.

A major limitation of HVV is the inability to perform fundoscopy or assess for a relative afferent pupillary defect. If the patient has significant new vision loss, the risks of an in-person examination during the pandemic should be weighed against the importance of timely identification of the cause of the vision loss with respect to treatment.

#### Cranial Nerves 3, 4, and 6

With the patient’s face centered and close to the camera, observe for pupillary asymmetry, ptosis, ocular alignment, or abnormal eye movements such as nystagmus. To test ocular motility, ask the patient to look in the nine cardinal positions of gaze, and it may be necessary to ask the patient to manually lift their eyelids during downgaze. Ask the patient to briefly pause at each gaze position looking for nystagmus. Horizontal saccades can be tested by asking the patient to alternate their gaze between the top right and left corners of the computer screen. Similarly, vertical saccade can be tested by asking the patient to alternate their gaze between just above the computer monitor and just below it.

#### Cranial Nerve 5

Inspect for temporalis atrophy and ask the patient to open their mouth, looking for jaw deviation. If there is a complaint of facial sensory disturbance, the patient can check sensation over the sensory distributions of the trigeminal nerve side-to-side with a tissue or something cold (e.g., ice pack).

#### Cranial Nerve 7

Observe baseline facial symmetry, including reduced movement on one side of the face and flattening of the nasolabial fold. Ask the patient to raise their eyebrows, close their eyes tight, purse their lips, show their teeth, and puff out their cheeks.

#### Cranial Nerve 8

Hearing cannot be formally assessed, and unilateral hearing loss cannot be confirmed through video. The examiner can inquire about hearing during the history.

#### Cranial Nerves 9 and 10

During the history, note any speech abnormality (nasality, scanning, and spastic dysarthria), and ask patient to phonate (“pa,” “ta,” and “ka”). Depending on video quality and lighting, ask the patient to open their mouth and observe soft palate elevation.

#### Cranial Nerve 11

Ask the patient to expose the neck and shoulders, evaluating for atrophy of the sternocleidomastoid and trapezius muscles. Have the patient look to the right and then the left and then to shrug their shoulders to test muscle activation.

#### Cranial Nerve 12

Depending on video quality, observe the tongue in the mouth, looking for fasciculations or atrophy. Ask the patient to protrude the tongue, and move the tongue side to side rapidly.

### Motor Examination

Please refer to Supplementary Video 3 for demonstration of a screening virtual motor examination.

Ideally, the patient should be instructed to wear shorts and t-shirt under their clothes and then change at this portion of the exam. Expose and inspect relevant areas of the upper and lower extremities (depending on the clinical concern) for muscle bulk and fasciculations (dependent on video resolution). Observe the patient with the arms at rest and then raise for abnormal movements (tremor, myoclonus, chorea, dystonia, or others). Further details of an examination for various movement disorders are beyond the scope of this document, but some aspects are noted in Table 3.

Subtle signs of pyramidal weakness can be detected by assessing for pronator drift, using the forearm rolling test, and testing of fine finger movements (tapping thumb with index finger repetitively or with each finger alternatively).

On power examination in the upper limbs at least antigravity strength can be noted by observing arm abduction, extension and flexion at the elbows, wrist and finger extension (with forearm pronated), wrist and finger flexion (with forearm supinated), finger abduction, thumb abduction, and thumb extension.

For the lower limbs, position the camera downward. While seated, ask the patient to raise each knee off the chair, to straighten the knees one at a time (knee extensors), and to dorsiflex and then plantarflex each foot. With arms crossed across the chest, ask them to stand up (requires at least Medical Research Council (MRC) grade 4/5 strength of proximal lower limb muscles). Note that the inability to perform this maneuver can also be due to other disorders that do not cause motor weakness, including Parkinsonian disorders. The patient can be asked to do 1–10 squats, to assess for fatigability or subtle proximal leg weakness. The patient should be asked to perform heel raises (dorsiflexion strength), and then do single foot toe raises (plantarflexion strength). They can also be asked to stand and hop on each leg. Axial muscle strength can be tested by asking the patient to perform sit-ups.

Tone, reflexes, and plantar responses cannot be formally assessed.

### Sensory Examination

Please refer to Supplementary Video 4 for demonstration of a screening virtual sensory examination.

The sensory examination cannot be formally performed. The specific regions to test and compare will depend on the reason for referral and patient’s sensory complaints (e.g., assessing a specific peripheral nerve distribution, or for length-dependent abnormalities). For a practical screening exam, one could ask the patient to use a nearby household item to compare light touch or temperature on the index fingers of both hands and the top of the big toes. Examples of household items would include a tissue or something cold (an ice pack or a cooled spoon placed under running water). To test for sensory ataxia, the patient can raise their arms, close their eyes, and touch their nose with each index finger separately.

### Coordination

Please refer to Supplementary Video 5 for demonstration of a screening virtual coordination examination.

If a family member or other third party is present, they can be asked to hold their finger out for standard finger-to-nose testing. If unavailable, ask the patient to hold an object (such as a pen) outstretched in front of them with one hand, and then with the contralateral index finger perform finger-to-object movements and then repeat on the contralateral side. Alternatively, the patient can be asked to hold their arms outstretched in front of them, and then touch their nose with each index finger. Rapid alternating movements can be checked as usual. Bradykinesia testing can be performed by having the patient perform finger taps and palm opening and closing maneuvers in both arms observing for any fatigability. Heel-to-shin testing can be performed by asking the patient to put each leg on a stool or ottoman and performing the maneuver. The patient can also be asked to perform foot tapping and heel stomping.

### Gait

Please refer to Supplementary Video 6 for demonstration of a screening virtual gait examination.

There may be limitations based on camera setup or size of the room. Observe stance at rest and with the feet together. Ask the patient to walk in two directions from one side of the room to the other and then walk away from the camera, turn around, and walk back to the camera. Ask the patient to attempt five steps in either direction before turning around. Tandem gait can be assessed similarly. It is not recommended to perform the Romberg test given the risk of the patient falling without the examiner to catch them.

### Other Maneuvers

Scapular winging can be assessed through video. The Phalen maneuver can also be performed (for carpal tunnel syndrome), as can be a spontaneous Spurling sign (tilt their head slightly to the symptomatic side and back) for cervical radiculopathy.

## Conclusions

In this article and the accompanying videos, we demonstrate how a neurological examination can be performed virtually, including some practical guidance. Our hope is that this information will increase the confidence of neurologists (as well as internists or general practitioners) in assessing neurological complaints through HVV. Certain neurological consultations are less amenable to a virtual examination, such as concern for myelopathy or cauda equina syndrome in which formal strength, reflexes, appendicular and rectal tone, and saddle anesthesia are critical maneuvers that cannot be performed virtually. It is important to reiterate that if there is concern about a possible urgent diagnosis, then the patient should be either arranged to be seen in-person or directed to seek urgent in-person medical attention.

The quality of each individual patient–physician virtual assessment is unique and dependent on a multitude of factors including the primary neurological complaint, the quality of the technology used, the ability of the patient to manipulate the camera appropriately and follow instructions, and the presence of a third party to assist with the examination. Regardless, the VNE is a powerful tool that has the potential to help deliver care to those who would otherwise be without timely access to neurological consultation and follow-up. This resource is meant to serve as a guide for clinicians to maintain high-quality neurological care during the COVID-19 pandemic and beyond.

## Supplementary material

For supplementary material accompanying this paper visit http://dx.doi.org/10.1017/cjn.2020.96. click here to view supplementary material
